# Determinants of Antiretroviral Treatment Adherence Among Young Mothers Living with HIV: The Role of Early Motherhood

**DOI:** 10.1007/s10461-025-04896-4

**Published:** 2025-10-15

**Authors:** Siyanai Zhou, Christina Laurenzi, Janke Tolmay, Camille Wittesaele, Nontokozo Langwenya, Alice Armstrong, Laurie Gulaid, Elona Toska

**Affiliations:** 1 Centre for Social Science Research, University of Cape Town, Cape Town, South Africa; 2 Institute for Life Course Health Research, Department of Global Health, Stellenbosch University, Tygerberg, South Africa; 3 Department of Social Policy and Intervention, University of Oxford, Oxford, UK; 4 Department of Infectious Disease Epidemiology, The London School of Hygiene & Tropical Medicine, London, UK; 5 UNICEF Eastern and Southern Africa Regional Office, Nairobi, Kenya; 6 Nuffield College, University of Oxford, Oxford, UK

## Abstract

Adolescent girls living with HIV are at higher risk of poor HIV treatment outcomes than older women living with HIV. However, little is known about how age at first birth influences their adherence to antiretroviral treatment (ART). Using South African data from a cross-sectional study of 311 young mothers living with HIV (*N* = 311, mean age 19.7 ± 1.92 years, follow-up = 2017–2018), we examined the association between age at first birth (< 18 versus 18+ years), all hypothesized factors and self-reported past-week ART adherence, adjusting for known covariates. We computed adjusted probability estimates conditional on the presence of none, one, or all factors significantly associated with ART adherence in the final regression model. Overall, 45% (*N* = 140) of participants had their first birth before the age of 18. ART adherence rates differed significantly by age at first birth (< 18 years: 67.9% vs. 18+ years: 80.1%, *p* = 0.013). Age-at-first birth (< 18 years) (aOR 0.45, 95% CI 0.26–0.78, *p* = 0.005) and internalized HIV stigma (aOR 0.33, 95% CI 0.17–0.62, *p* = 0.001) were associated with lower odds of past-week adherence, while psychosocial support (aOR 2.39, 95% CI 1.20–4.74, *p* = 0.013) and availability of a caregiver for the participant (aOR 2.74, 95% CI 1.37–5.52, *p* = 0.005) were associated with higher odds of past-week adherence. Combined psychosocial support and caregiver presence improved ART adherence among young mothers, with stronger effects in those reporting internalized HIV stigma. Efforts to support young mothers to improve their adherence to ART are urgently needed at scale, including strategies to reduce the impact of HIV-related stigma, including family psychosocial support.

**Supplementary Information:**

The online version contains supplementary material available at 10.1007/s10461-025-04896-4.

##  Introduction

Adolescent girls in Eastern and Southern Africa are at high risk of HIV, unintended pregnancy, and early motherhood, risks which can intersect to adversely affect their health and well-being during a pivotal life stage [1, 2]. In South Africa, 12.3% of girls ages 15–19 are living with HIV, and 16% of all girls aged 15–19 become pregnant before the age of 20 [3, 4]. Despite widely available health services in South Africa, young women also face stigmatization in health settings, limiting their access to HIV and sexual and reproductive health (SRH) care [5, 6]. The social and epidemiological factors that often drive HIV acquisition and unintended pregnancies in adolescents—poverty, food insecurity, poor mental health, and violence—disrupt engagement along the HIV care continuum, contributing to lower rates of testing, initiation of antiretroviral therapy (ART), and retention in care [7]. Young mothers under age 24 who are living with HIV are more likely to have poorer treatment adherence and higher rates of vertical transmission than older mothers [8, 9]. Additionally, adolescent and young mothers have been found to be more likely to miss healthcare visits, including the 18-month postpartum follow-up visit compared to mothers older than 24 years [10].

Despite these well-understood risks, little is known about the experiences of young mothers living with HIV who give birth before the age of 18—a growing cohort since the COVID-19 pandemic [11]. This group is often omitted from research and programming for ethical, legal, methodological, or stigma-related reasons [12, 13]. Research involving young mothers living with HIV is often conducted within broader studies of mothers of all ages or without specific attention to HIV status—or they may be excluded altogether. This oversight neglects critical developmental and psychosocial factors affecting their clinical outcomes [14]. While adolescence can present opportunities for establishing healthy behaviors, young people who experience certain adversities can also struggle to achieve and maintain healthy trajectories [15]. As young mothers living with HIV navigate critical life transitions—HIV diagnosis, pregnancy, birth and motherhood—they are also likely to face extremely difficult decisions about how to care for themselves and their children. At a time of increasing health and psychosocial needs, many young mothers experience isolation and stigma, which can interrupt their access to maternal and infant health care and their ability to be initiated on HIV treatment and/or remain retained in HIV care [7, 16].

Retention in care is critical to long-term HIV treatment outcomes, yet there is limited research on patterns and factors associated with retention in HIV care among young mothers. Developing responsive interventions for young mothers living with HIV relies on a clearer understanding of the factors that shape their experience with adherence, to identify potential factors that can be effectively targeted. Given the complex social, psychological, and structural factors that shape HIV outcomes for this priority group, we set out to analyze the effect of the mother’s age at first birth on adherence to ART, as well as explore other factors shaping adherence among adolescent mothers, using a large cohort study from South Africa.

##  Methods

### Study Setting and Period

The study uses cross-sectional data collected from young mothers recruited from urban and rural locations in two health districts in the Eastern Cape Province, South Africa, in 2017–2018 [17]. The Eastern Cape has one of the highest rates of adolescent pregnancy in the country, with over 60 births per 1000 girls reported among 10–19-year-olds in 2021 [4]. In 2022, the selected study districts (Amathole and Buffalo City) had some of the highest HIV prevalence rates—34% and 32% respectively—among women in antenatal care nationally [18].

##  Study Design and Sample

In total, 1,046 adolescent girls and young women aged 10–24 years were recruited in the two districts. Adolescent girls and young women were eligible for inclusion in the study if they were aged 10–24 at recruitment and had had their first child before the age of 20 [19]. Recruitment was conducted through multiple channels developed with local experts and an advisory group of young mothers to minimize recruitment bias and expand reach to a sample of participants that was not biased by access to services. These channels included recruitment via health facilities, maternity obstetric units, randomly selected secondary schools, referrals by social workers and service providers, and community referrals by young mothers themselves [17]. Between 95 and 98% of young mothers identified through each channel were successfully enrolled in the study [17].

### Data Collection Procedures

Assisted by trained interviewers, participants completed a structured questionnaire on a tablet about their multi-dimensional experiences of health, HIV, and healthcare access. The questionnaire included items on participant sociodemographic characteristics and living arrangements, and retrospective questions aiming to capture events at the time of pregnancy. Interviews were conducted in private spaces, at participants’ homes or another preferred location. All questionnaires were available in English and Xhosa. Our analyses focus on data from 311 young mothers living with HIV—29.7% of the total sample. HIV status among all participants was ascertained through medical records, including either a confirmed HIV-positive test result, CD4 count or viral load (VL) at treatment initiation prior to the interview [20]. A robust referrals process was developed to respond to any concerns that were flagged during the interview process—including but not limited to social issues, mental distress, violence, and suicidality.

### Measures

*Outcome*: Past-week ART adherence was defined based on self-report of currently taking ART and not having missed any doses in the past 7 days (including weekdays and weekends), adapted from the Patient Medication Adherence Questionnaire [21]. If the participant reported missing any dose in the past 7 days or currently not taking ART (i.e., defaulting) [22], we classified them as non-adherent. Past-week ART adherence was coded as 1, and non-adherence as 0. Previous analyses of this measure among adolescents living with HIV in the study area have found strong associations between this self-reported past-week ART adherence measure and VL outcomes [23].

*Covariates* included participants’ age (categorized into three groups: < 18, 18–21, and 21 + to minimize multicollinearity with age at first child); residence (urban/rural); housing type (informal/formal); relationship status; and food insecurity, defined by combining (1) reports of not having enough food for the entire week and (2) not affording three daily meals at home. Mode of maternal HIV acquisition (recent/perinatal) was computed via an algorithm based on the age of ART initiation, validated with self-reported data such as the age of first sex, orphanhood cause and experiences of sexual assault [24]. Parity was defined based on the self-reported total number of births. *Time since birth of the first child* (in years) was computed as a difference between the mother’s age at the time of the interview and the age at birth of the first child (median 1.46 years, IQR 0.21–2.69). Parity was defined based on the self-reported total number of births.

*Hypothesized factors*: Our target variable, *age at first birth*, was defined as a binary measure coded 1 if the participant had their first birth before age 18, and 0 otherwise. For *age at first birth*, a cut-off of 18 years was chosen based on the age of majority in South Africa, as well as a benchmark for capacity [25]. We explored additional key factors, including *mental health symptoms*, *internalized HIV stigma* and *perceived HIV stigma*, *caregiver presence*, *psychosocial support*, and *positive future aspirations*. These factors were selected based on evidence of their impact on ART adherence among adolescents and young people living with HIV [26]. Table 1 shows these additional factors, alongside their definitions, measurement scales and coding.

**Table 1:** Description of key hypothesized factors associated with ART adherence

Variable	Description and coding
Any mental health symptoms	A composite measure based on the adolescent’s experiences of any symptoms of depression, anxiety, and suicidality. Depression symptoms (past two weeks) were measured using the Child Depression Inventory (CDI-S) short form, a widely used 10-item version [27] validated in other South African studies [28]. Anxiety symptoms (past month) were measured using the Children’s Manifest Anxiety Scale-Revised (RCMAS), a 14-item abbreviated version. Suicidality was defined based on suicidal thoughts and behavior in the past month. Participants responded to a scale of five items that asked if, in the past month, they (1) wished they were dead, (2) wanted to hurt themselves, (3) thought about killing themselves, (4) thought of a way to kill themselves, and (5) tried to kill themselves [29]. Participants who responded ‘yes’ to any of the five items were coded as experiencing suicidality. All measures performed well in this sample [30]
Internalized HIV stigma	Internalized HIV stigma was based on adolescents’ experiences of any of the items from the ALHIV Stigma Scale (ALHIV-SS), developed in collaboration with adolescents living with HIV in South Africa, with strong internal consistency in a large cohort of ALHIV in the study context [31]
Perceived stigma	Perceived stigma was defined based on responses to the following items ‘*People in the community think that a person with HIV is disgusting’* and ‘*People in my community think that HIV is a punishment from God or ancestors*.’ We coded responses as perceived stigma if the participant responded ‘sometimes’ or ‘most of the time’ to either item
Caregiver presence	Caregiver presence was defined as a binary indicator of living with a caregiver, parents, or grandparents versus others
Psychosocial support	*Psychosocial support* was measured using the Medical Outcomes Study Social Support Survey (MOS-SSS) scale’s seven support items [32]. These included items on availability of someone to *(1) listen to you; (2) give you good advice; (3) share worries with; (4) turn to for suggestions; (5) help if confined in bed; (6) take you to the doctor;* and *(7) prepare meals*. Respondents were asked to rate how often each type of support was available when they needed it and chose one of the three options ranging from ‘never’ (0) to ‘always’ (2). Participants who rated ‘always’ on all seven items were coded as accessing higher levels of psychosocial support. The psychosocial support scale has consistently demonstrated strong psychometric properties, with a Cronbach’s alpha of 0.94 and robust construct validity
Positive future aspirations	*Positive future aspirations* were measured using 6 items, which included their thoughts on the future and how likely or unlikely the following were: (1) *I will have a job*, (2) *I will have a house*, (3) *I will be able to afford food*, *clothing and shelter for myself*, (4) *I will be able to take care of my health and keep strong and well*, (5) *I will have a happy relationship with a long-term partner such as a husband or wife*, and (6) *I will have happy and healthy children.* Participants’ responses ranged from “very unlikely” to “very likely”, and if the participant responded ‘very likely’ to all 6 items, then we coded this as positive future aspirations

### Statistical Analysis

First, descriptive characteristics of participants, overall and by past-week self-reported ART adherence, were summarized using means, standard deviations, and frequencies. We calculated differences in rates of past-week adherence by participant characteristics, including age at first birth (< 18 years) and other important factors, using t-tests for continuous variables and Chi-square tests for categorical variables. Second, multivariable logistic regression analysis was used to examine (i) the association between past-week ART adherence and age at first birth (< 18 years), and (ii) the association between past-week ART adherence and all key factors, including age at first birth (< 18 years). For the second part of the analysis (ii), stepwise multivariate regression models were conducted, with the first model including all covariates, age at first birth, and all key factors. The second model included only factors that were significant at a 10% level (*p* < 0.1). The third and final model included only factors significant at the 5% level (*p* < 0.05). All models were adjusted for the following confounders: participants’ age, mode of HIV acquisition, urban/rural residence, informal housing, relationship status, and food insecurity. Finally, we estimated adjusted predicted probabilities to compare past-week ART adherence rates by age at first birth across different combinations of all factors significantly associated with ART adherence. We conducted two sets of sensitivity analyses: first, using a cutoff of 19 years for age at first birth, and second, restricting the sample to participants older than 18 years. Multicollinearity was assessed for all key factors and covariates using Spearman’s correlation and variance inflation factors. All analyses were conducted in Stata 18 [33].

## Results

### Sample characteristics

Overall, 311 adolescent and young mothers living with HIV who had a child before the age of 20 were included in this analysis. Participants were 19.7 years old on average (SD: 1.93), and 45% (*n* = 140) had first given birth before age 18. One-quarter of participants (*n* = 76) lived in rural communities, one-third (*n* = 87) lived in informal housing (constituting a shack on a plot or backyard), and about one-third (*n* = 97) experienced past-week food insecurity. Nearly 12% of young mothers (*n* = 37) had vertically-acquired HIV, and 73.1% (*n* = 229) reported being in a romantic relationship (Table 2). About three-quarters (74.6%; *n* = 232) of the group reported past-week ART adherence. In a bivariate analysis comparing rates of ART adherence, participants who had their first birth before age 18 (*p* = 0.013), had a longer time since their first birth (*p* = 0.002), reported perceived HIV stigma (*p* = 0.024) and/or internalized HIV stigma (*p* < 0.001), and reported any mental health symptoms (*p* = 0.007) were less likely to report past-week adherence. Participants who reported positive future aspirations (*p* = 0.006), lived with a caregiver (*p* = 0.002), and received social support from family (*p* < 0.001) were significantly more likely to report past-week adherence (Table 2).

**Table 2:** Socio-demographic characteristics, age at first birth and key factors by past-week self-reported ART adherence

	Total (*N* = 311)	Past-week adherence (*N* = 232)	Past-week non-adherence (*N* = 79)	*p* value
Hypothesized factors (*n*, %)				
Age at first birth (< 18): n (%)	140 (45.0)	95 (40.9)	45 (57.0)	**0.013**
Any mental health symptoms: n (%)	49 (15.8)	29 (12.5)	20 (25.3)	**0.007**
Internalized HIV stigma: n (%)	62 (19.9)	33 (14.2)	29 (36.7)	**< 0.001**
Perceived stigma: n (%)	70 (22.5)	45 (19.4)	25 (31.6)	**0.024**
Live with caregiver: n (%)	266 (85.5)	207 (89.2)	59 (74.7)	**0.002**
Social support: n (%)	260 (83.6)	205 (88.4)	55 (69.6)	**< 0.001**
Positive future aspirations: n (%)	226 (72.7)	178 (76.7)	48 (60.8)	**0.006**
Covariates				
Age group at baseline: n (%)				0.774
< 18 years	32 (10.3)	24 (10.3)	8 (10.1)	
18–21 years	213 (68.5)	161 (69.4)	52 (65.8)	
> 21 years	66 (21.2)	47 (20.3)	19 (24.1)	
Rural residence: n (%)	76 (24.4)	59 (25.4)	17 (21.5)	0.480
Informal housing: n (%)	87 (28)	58 (25)	29 (36.7)	**0.045**
In a relationship: n (%)	229 (73.6)	168 (72.4)	61 (77.2)	0.401
Food insecurity: n (%)	97 (31.2)	63 (27.2)	34 (43.0)	**0.008**
Vertically-acquired HIV: n (%)	37 (11.9)	23 (9.9)	14 (17.7)	0.064
Parity: mean (SD)	1.23 (0.48)	1.33 (0.61)	1.19 (0.42)	**0.030**
Time since first birth (years): mean (SD)	1.81 (1.78)	1.62 (1.75)	2.33 (1.76)	**0.002**

Table 3 shows an assessment of the distribution of sociodemographic factors, key factors, and ART adherence by age at first birth. Rates of ART adherence were significantly different for the two groups (67.9% versus 80.1%, *p* = 0.013). There were no differences in reporting key baseline factors across the two groups. Participants who had their first birth before the age of 18 were more likely to be younger, have a longer time since their first birth, and were less likely to report being in a romantic relationship.

**Table 3:** Differences in sociodemographic factors, key factors, and ART adherence by age at first birth

	Total	Age at first birth		
	*N* = 311	< 18 years (*N* = 140)	18 + years (*N* = 171)	*p* value
Hypothesized factors				
Past-week adherence: n (%)	232 (74.6)	95 (67.9)	137 (80.1)	**0.013**
Any mental health symptoms: n (%)	49 (15.8)	20 (14.3)	29 (17.0)	0.52
Internalized HIV stigma: n (%)	62 (19.9)	26 (18.6)	36 (21.1)	0.59
Perceived stigma: n (%)	70 (22.5)	31 (22.1)	39 (22.8)	0.89
Live with caregiver: n (%)	266 (85.5)	121 (86.4)	145 (84.8)	0.68
Social support: n (%)	260 (83.6)	118 (84.3)	142 (83.0)	0.77
Positive future aspirations: n (%)	226 (72.7)	98 (70.0)	128 (74.9)	0.34
**Covariates**				
Age				**< 0.001**
Min–Max	14–25	14–23	18–25	
Median, IQR	20 (19–21)	20 (18–20)	20 (19–22)	
Age group at baseline: n (%)				**< 0.001**
< 18 years	32 (10.3)	32 (22.9)	0 (0)	
18–21 years	213 (68.5)	90 (64.3)	123 (71.9)	
> 21 years	66 (21.2)	18 (12.8)	48 (28.1)	
Rural residence: n (%)	76 (24.4)	34 (24.3)	42 (24.6)	0.96
Informal housing: n (%)	87 (28.0)	42 (30.0)	45 (26.3)	0.47
In a relationship: n (%)	229 (73.6)	95 (67.9)	134 (78.4)	**0.036**
Food insecurity: n (%)	97 (31.2)	42 (30.0)	55 (32.2)	0.68
Parity: mean (SD)	1.23 (0.48)	1.36 (0.58)	1.12 (0.34)	**< 0.001**
Time since first birth (years): mean (SD)	1.81 (1.78)	2.62 (2.07)	1.14 (1.13)	**< 0.001**
Vertically-acquired HIV: n (%)	37 (11.9)	19 (13.6)	18 (10.5)	0.41

### Factors Associated with Past-Week ART Adherence Among Young Mothers

**Fig. 1:**
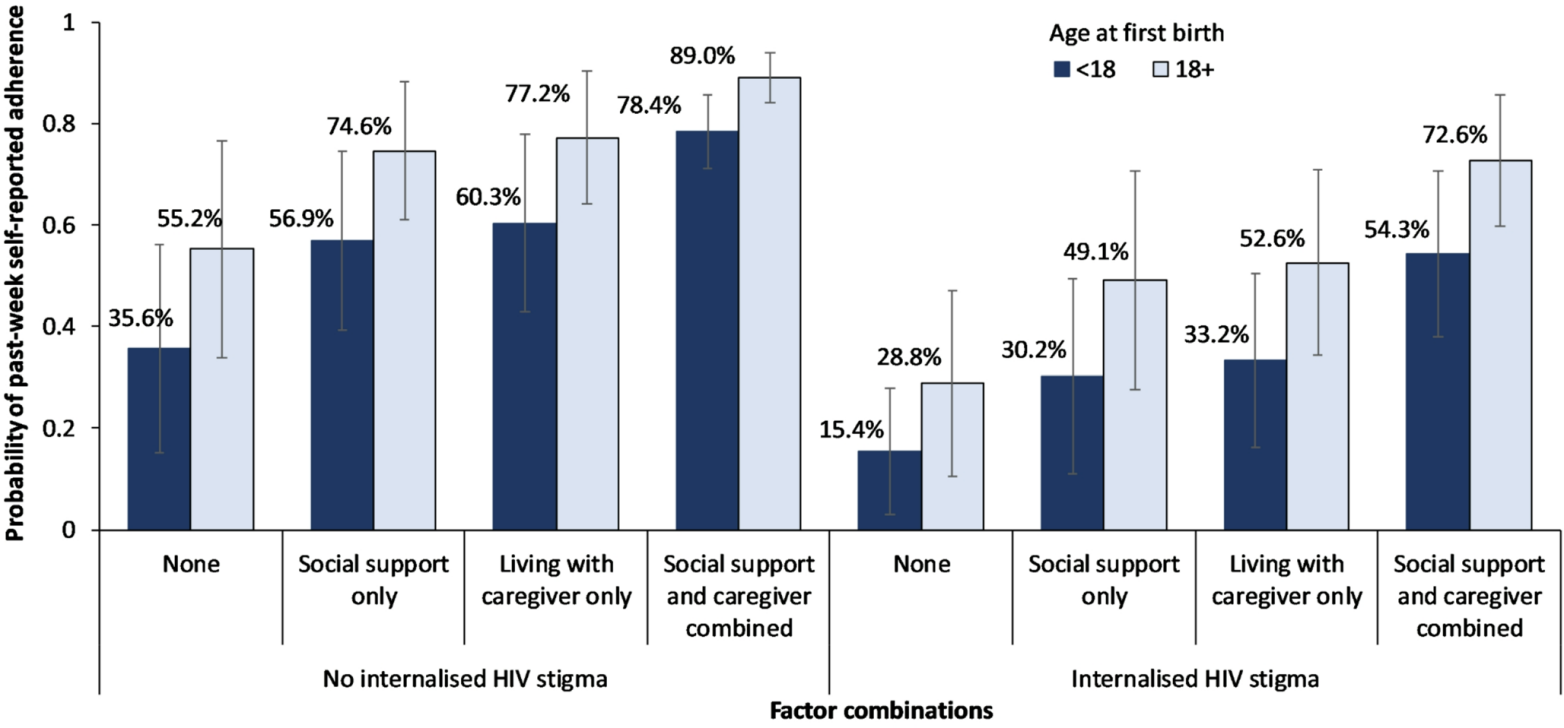
Adjusted predicted probabilities of past-week adherence by age at first birth (< 18 versus 18 + years) among young mothers living with HIV

In multivariable analyses (Table 4, Model 1) with one predictor and controls, age at first birth was associated with past-week ART adherence. Respondents who had their first birth before the age of 18 years were likelier to report lower past-week ART adherence (aOR = 0.45, 95% CI 0.24–0.84, p value = 0.012). Food insecurity (aOR = 0.49, 95% CI 0.28–0.87, p value = 0.015) was also associated with lower odds of past-week ART adherence. In multivariate analyses with all hypothesized predictors, four factors were associated with past-week ART adherence (Table 4, Model 4). Participants who had a child before the age of 18 were less likely to report past-week ART adherence (aOR = 0.45, 95% CI 0.26–0.78, *p* = 0.005), and those who experienced any HIV-related internalized stigma were less likely to report past-week ART adherence (aOR = 0.33, 95% CI 0.17–0.62, *p* = 0.001). Participants who reported living with a caregiver were more likely to report past-week ART adherence (aOR = 2.74, 95% CI 1.36–5.52, *p* = 0.005) while those who received family-related social support were more likely to report past-week ART adherence (aOR = 2.39, 95% CI 1.20–4.73, *p* = 0.013). A sensitivity analysis using a cutoff of 19 years for age at first birth yielded similar results (Table S1 in Supplementary Material). An additional sensitivity analysis restricted to participants over the age of 18 yielded findings consistent with those from the full sample (Table S2 in Supplementary Material). Based on the final Model 4, we estimated average marginal probabilities for the combinations of the four factors (Fig. 1). Assuming that the distribution of all other factors remained the same among the respondents, for those who had a child before the age of 18, living with a caregiver while also receiving social support was associated with greater improvements in past-week ART adherence regardless of HIV stigma experience: +39% for those who experienced internalized HIV stigma and + 42.8% for those who did not (Fig. 1). For respondents who had a child at age 18 and above, living with a caregiver while also receiving social support was associated with greater improvements in past-week ART adherence for those who reported internalized HIV stigma (+ 43.8%) compared to those who did not (+ 33.8%).

**Table 4:** Factors associated with past-week ART adherence among young mothers living with HIV in South Africa (*N* = 311)

	Past-week ART adherence							
	Model 1 (*N* = 311)		Model 2: Full model (*N* = 311)		Model 3 (*p* < 0.10) (*N* = 311)		Model 4 (*p* < 0.05) (*N* = 311)	
	aOR (95% CI)	*p* value	aOR (95% CI)	*p* value	aOR (95% CI)	*p* value	aOR (95% CI)	*p* value
Main factors								
Age at first birth (< 18)	0.45 (0.24–0.84)	**0.012**	0.34 (0.17–0.68)	**0.002**	0.38 (0.21–0.69)	**0.002**	0.45 (0.26–0.78)	**0.005**
Any mental health symptoms	–	–	0.77 (0.35–1.70)	0.522				
Internalized HIV stigma	–	–	0.34 (0.16–0.70)	**0.003**	0.32 (0.17–0.61)	**0.001**	0.33 (0.17–0.62)	**0.001**
Perceived stigma	–	–	0.88 (0.43–1.80)	0.733				
Live with a caregiver	–	–	2.19 (1.04–4.61)	**0.039**	2.74 (1.35–5.55)	**0.005**	2.74 (1.36–5.52)	**0.005**
Social support	–	–	2.11 (1.01–4.41)	**0.047**	2.26 (1.12–4.56)	**0.022**	2.39 (1.20–4.73)	**0.013**
Positive future aspirations	–	–	1.37 (0.72–2.62)	0.342				
Covariates								
Age group at baseline								
< 18 years (ref)	1		1					
18–21 years	0.64 (0.24–1.69)	0.370	0.49 (0.17–1.45)	0.198	0.54 (0.20–1.49)	0.234		
> 21 years	0.45 (0.14–1.45)	0.181	0.33 (0.09–1.19)	0.090	0.36 (0.11–1.16)	0.086		
Rural residence	1.39 (0.73–2.64)	0.316	1.52 (0.75–3.09)	0.241				
Informal housing	0.62 (0.35–1.09)	0.097	0.65 (0.35–1.22)	0.180				
In a relationship	0.72 (0.38–1.35)	0.298	0.67 (0.33–1.34)	0.259				
Food insecurity	0.50 (0.28–0.87)	**0.015**	0.61 (0.33–1.11)	0.107				
Parity	0.87 (0.48–1.57)	0.634	1.11 (0.57–2.14)	0.763				
Vertically acquired HIV	0.47 (0.21–1.02)	0.054	0.49 (0.21–1.13)	0.095	0.48 (0.21–1.06)	0.069		

*aOR* adjusted odds ratio, *95% CI* confidence interval

Time since first birth was removed from the analysis due to collinearity, as it was highly correlated with other variables, to improve the accuracy and interpretability of the model

## Discussion

.

Our analysis presents compelling evidence of persistent challenges in ART adherence among young mothers living with HIV. To date, there has been limited data on patterns of adherence among the youngest mothers living with HIV, and the factors associated with these patterns [34, 35]. Because health behaviors tend to cluster, understanding challenges to retention in care may influence efforts to improve related HIV and SRH outcomes for this group, such as ART adherence, early infant testing and diagnosis, and maternal and child health service uptake [36]. In our data, young mothers who had their first birth before 18 were less likely to report past-week adherence, as were those participants experiencing food insecurity, living in informal housing, and lacking social support. Poverty-related material needs have been associated with non-adherence in other studies [7], including with younger populations, while psychosocial factors that include both mental health symptoms and diverse forms of stigma, have also been shown to have profound effects on the ability to adhere to ART [37, 38]. We found that young mothers reporting worse mental health symptoms and internalized HIV stigma were less likely to report past-week adherence.

Our findings also draw out key considerations surrounding maternal age at first birth with other factors associated with ART adherence, which have not been examined in previous studies. In our final model, maternal age at first birth, internalized stigma, caregiver presence, and psychosocial support all emerged as significant in shaping adherence. The compounding effects of internalized HIV stigma and limited social support can complicate efforts to achieve consistent adherence—indicating that beyond linkage to services, the most vulnerable adolescents living with HIV may struggle to achieve high adherence rates without additional care or specific attention to internalized stigma.

Adolescence is a time of significant biological, behavioral and psychological change, and while many adolescents may struggle to have consistent adherence, young mothers who are also living with HIV face additional challenges [39]. Undergoing transitions to motherhood and adult HIV care, while facing changes in social and family lives, may elevate stressors for young women at this pivotal life stage, inhibiting adherence [40]. In communities with high rates of both early motherhood and HIV, these adherence challenges pose significant risks for young mothers’ morbidity and mortality. They also may threaten the significant gains made in reducing and preventing new cases of HIV, especially among partners and infants of young mothers. These findings also emphasize the importance of monitoring child health and HIV outcomes in this transition period. Children born to young mothers living with HIV have poorer access to early infant HIV testing [10] and three times higher risk of perinatal HIV acquisition compared to children of adult mothers living with HIV [8, 36]. These disparities reiterate the added vulnerabilities of younger mothers and highlight the need for more focused attention on adolescents in healthcare and educational spaces.

Our analysis reveals possible entry points for targeting interventions to improve adherence, especially for younger mothers experiencing overlapping adversities. Internalized HIV-related stigma emerged consistently alongside non-adherence, superseding any other form of enacted or perceived stigma about HIV, or mental health symptomatology. From a developmental perspective, adolescents living with HIV may experience both neurocognitive and psychosocial challenges during their adolescence, heightening the stigma that they experience [41]. Young mothers often experience these challenges alongside amplified social disempowerment, structural disadvantage, and life course disruptions that accompany motherhood, increasing the likelihood of internalized HIV stigma. However, the combined effects of social support and caregiver presence improved the probability of adherence by nearly 40% among younger mothers experiencing internalized HIV stigma in our analysis. This finding suggests that beyond directly targeting internalized HIV stigma, bolstering supportive systems for young mothers may yield similar benefits to adherence.

In the shorter term—and during the critical peripartum period—interventions focused on strengthening social support may counteract the negative effects of internalized stigma and broaden adolescent mothers’ networks and ability to navigate towards supportive resources. Integrating mental health promotion and prevention for adolescents living with HIV [42, 43], especially young mothers [44] is essential. Similarly, providing stepped-care approaches for those experiencing multiple adversities can help stem challenges that disrupt adherence. Promising layered intervention approaches include peer support and “youth champion” programs [45], including the Young Mentor Mother Programme in Zimbabwe [46], as well as individualized approaches that can enhance self-care and empower young people [47]. While caregiving arrangements may vary, young mothers are likely to benefit from strategies that adopt intergenerational approaches and tap into family constellations to incorporate diverse support systems and relationships. Where available, family-based support or counselling can support young mothers and their infants.

Strategies to support young mothers should also consider the places where they can be best reached. Key staff and peers in schools, health facilities, and communities can help to address the critical, tangible needs that adolescent girls face during their pregnancies. While perceived HIV stigma did not emerge as salient in our analysis, nearly 1 in 4 participants reported such experiences. Adolescents who become pregnant and drop out of school, or are otherwise stigmatized, are at heightened risk of disengaging from social support networks and healthcare at a particularly high-risk time [48]. School dropout and expulsion remain widespread, despite South African policies to support enrollment during pregnancy and re-enrollment after delivery [49, 50]. The study’s multi-pronged recruitment strategy revealed the extent to which many young mothers are no longer in school, and similarly, some individuals may require enhanced support to re-engage in HIV care [51]. Our ongoing work in the study site with young mothers living with HIV and trusted adults in their support networks is framed to elevate their specific preferences and priorities. Ultimately, research and programming should continue to prioritize the needs of young mothers, their infants, and their broader networks to determine how to improve their health, well-being, and security.

To be best implemented, these recommendations rely on continued access to high-quality medication and the necessary psychosocial interventions that have been made possible through coordinated, multi-sectoral international development aid, with national and local government leadership [52]. Without sustained commitments to supporting the clinical and infrastructural resources essential to maintaining a high standard of HIV care, vulnerable populations, including young mothers, are likely to experience greater degrees of risk, threatening their health and their lives.

### Limitations

.

This study has a number of limitations. First, all data is self-reported, which may invite both recall bias and social desirability bias. To mitigate bias, we piloted the questionnaire with a Teen Advisory Group and employed well-trained, empathetic local interviewers, many of whom had been young parents themselves. Due to the study design, we considered the oldest living child as the firstborn. Second, the generalizability of these findings to other low- and middle-income settings remains unknown. However, the socioeconomic conditions, HIV prevalence rates, health systems, and structural drivers in the study area may resemble conditions across Southern Africa.

## Conclusions

Adherence to ART remains a persistent challenge for young mothers in South Africa, especially those who give birth before 18. Approaches to support this priority population should focus on identifying avenues to reduce internalized HIV stigma, fostering supportive home environments and relationships, and increasing sensitivity in health care provision. These interventions can be integrated into existing service delivery and programming to increase their reach and improve the health and wellbeing of young mothers and their children across the life course. Critically, programming relies on sustained financial and moral commitments and a broad conceptualization of the right to health in order to support the diverse maternal and child health needs of this population.

## Supplementary Information

Below is the link to the electronic supplementary material.

Supplementary Material 1

## Data Availability

Data is available upon request following the correct procedures. Data is not yet available open access as the study has not concluded; therefore, data is not fully anonymised.

## Code Availability

STATA do-files are available on request from co-authors.
