# Supplementary material for: Determinants of Antiretroviral Treatment Adherence Among Young Mothers Living with HIV: The Role of Early Motherhood
Source: AIDS Behav. 2025 Oct 15;30(3):713–22. doi: 10.1007/s10461-025-04896-4 (PMC12988881; doi:10.1007/s10461-025-04896-4)
Supplement: Supplementary file 1 — Supplementary Material 1 [file 10461_2025_4896_MOESM1_ESM.docx]

**Determinants of antiretroviral treatment adherence among young mothers living with HIV: the role of early motherhood**

Table of contents:

**Table S1**: Factors associated with past-week ART adherence among young mothers and young women living with HIV: A sensitivity analysis using a cutoff of 19 years for age at first birth (N=311)……………………………………………………………………………..2

**Table S2:** Factors associated with past-week ART adherence among young mothers and young women living with HIV: A sensitivity analysis, limiting the sample to those over age 18 (N=240)……………………………………………………………………………………3

**Table S1: Factors associated with past-week ART adherence among young mothers and young women living with HIV: A sensitivity analysis using a cutoff of 19 years for age at first birth** **(N=311)**

|  | **Past-week ART adherence** | | | | | |
| --- | --- | --- | --- | --- | --- | --- |
|  | **Full model**  **(N=311)** | | **Model 2 (p<0.10)**  **(N=311)** | | **Model 3 (p<0.05)**  **(N=311)** | |
| **Baseline factors** | **aOR (95%CI)** | **p-value** | **aOR (95%CI)** | **p-value** | **aOR (95%CI)** | **p-value** |
| Age at first birth (<19) | 0.41 (0.19-0.87) | **0.020** | 0.51 (0.27- 0.98) | **0.042** | 0.49 (0.26- 0.94) | **0.033** |
| Any mental health symptoms | 0.89 (0.41-1.91) | 0.762 |  |  |  |  |
| Internalized HIV stigma | 0.35 (0.17-0.73) | **0.005** | 0.35 (0.19- 0.66) | **0.001** | 0.35 (0.18- 0.65) | **0.001** |
| Perceived stigma | 0.86 (0.43-1.75) | 0.685 |  |  |  |  |
| Live with a caregiver | 1.99 (0.95-4.16) | 0.069 | 2.68 (1.33- 5.37) | **0.006** | 2.59 (1.29- 5.18) | **0.007** |
| Social support | 2.06 (0.99-4.27) | 0.052 | 2.23 (1.12- 4.44) | **0.022** | 2.36 (1.19- 4.66) | **0.013** |
| Positive future aspirations | 1.37 (0.72-2.60) | 0.331 |  |  |  |  |
| **Covariates** |  |  |  |  |  |  |
| Age group at baseline |  |  |  |  |  |  |
| <18 years (ref) | 1 |  |  |  |  |  |
| 18-21 years | 0.74 (0.27-2.04) | 0.558 |  |  |  |  |
| >21 years | 0.45 (0.13-1.57) | 0.210 |  |  |  |  |
| Rural residence | 1.55 (0.77-3.12) | 0.217 |  |  |  |  |
| Informal housing | 0.62 (0.34-1.16) | 0.134 |  |  |  |  |
| In a relationship | 0.74 (0.38-1.45) | 0.378 |  |  |  |  |
| Food insecurity | 0.66 (0.36-1.19) | 0.166 |  |  |  |  |
| Parity | 0.91 (0.49-1.71) | 0.776 |  |  |  |  |
| Vertically acquired HIV | 0.49 (0.22-1.13) | 0.094 | 0.56 (0.26- 1.22) | 0.147 |  |  |

**Table S2: Factors associated with past-week ART adherence among young mothers and young women living with HIV: A sensitivity analysis, limiting the sample to those over age 18 (N=240).**

|  | **Past-week ART adherence** | | | | | |
| --- | --- | --- | --- | --- | --- | --- |
|  | **Full model**  **(N=240)** | | **Model 2 (p<0.10)**  **(N=240)** | | **Model 3 (p<0.05)**  **(N=240)** | |
| **Baseline factors** | **aOR (95%CI)** | **p-value** | **aOR (95%CI)** | **p-value** | **aOR (95%CI)** | **p-value** |
| Age at first birth (<19) | 0.35 (0.17-0.71) | **0.004** | 0.40 (0.21-0.74) | **0.004** | 0.40 (0.21-0.74) | **0.004** |
| Any mental health symptoms | 0.72 (0.29-1.76) | 0.466 |  |  |  |  |
| Internalized HIV stigma | 0.27 (0.12-0.63) | **0.002** | 0.29 (0.15-0.60) | **0.001** | 0.29 (0.15-0.60) | **0.001** |
| Perceived stigma | 1.32 (0.56-3.11) | 0.526 |  |  |  |  |
| Live with a caregiver | 2.09 (0.93-4.68) | 0.074 | 2.15 (1.01-4.64) | **0.048** | 2.15 (1.01-4.64) | **0.048** |
| Social support | 1.98 (0.86-4.52) | 0.096 | 2.22 (1.03-4.81) | **0.042** | 2.22 (1.03-4.81) | **0.042** |
| Positive future aspirations | 1.30 (0.63-2.69) | 0.478 |  |  |  |  |
| **Covariates** |  |  |  |  |  |  |
| Age group at baseline |  |  |  |  |  |  |
| 18-21 years | 1 |  |  |  |  |  |
| >21 years | 0.76 (0.36-1.60) | 0.465 |  |  |  |  |
| Rural residence | 1.07 (0.51-2.26) | 0.860 |  |  |  |  |
| Informal housing | 0.78 (0.39-1.56) | 0.489 |  |  |  |  |
| In a relationship | 0.76 (0.35-1.67) | 0.500 |  |  |  |  |
| Food insecurity | 0.74 (0.37-1.46) | 0.381 |  |  |  |  |
| Parity | 1.14 (0.57-2.27) | 0.719 |  |  |  |  |
| Vertically acquired HIV | 0.49 (0.17-1.44) | 0.197 |  |  |  |  |
